# Motor Unit Properties of the First Dorsal Interosseous in Chronic Stroke Subjects: Concentric Needle and Single Fiber EMG Analysis

**DOI:** 10.3389/fphys.2018.01587

**Published:** 2018-12-03

**Authors:** Bo Yao, Cliff S. Klein, Huijing Hu, Sheng Li, Ping Zhou

**Affiliations:** ^1^Institute of Biomedical Engineering, Chinese Academy of Medical Sciences & Peking Union Medical College, Tianjin, China; ^2^Department of Physical Medicine & Rehabilitation, The University of Texas Health Science Center at Houston, Houston, TX, United States; ^3^TIRR Memorial Hermann Research Center, Houston, TX, United States; ^4^Guangdong Work Injury Rehabilitation Center, Guangzhou, China

**Keywords:** EMG, stroke, fiber density, motor unit action potential, muscle, skeletal, rehabilitation

## Abstract

The purpose of this study was to better understand changes in motor unit electrophysiological properties in people with chronic stroke based on concentric needle electromyography (EMG) and single fiber EMG recordings. The first dorsal interosseous (FDI) muscle was studied bilaterally in eleven hemiparetic stroke subjects. A significant increase in mean fiber density (FD) was found in the paretic muscle compared with the contralateral side based on single fiber EMG (1.6 ± 0.2 vs. 1.3 ± 0.1, respectively, *P* = 0.003). There was no statistically significant difference between the paretic and contralateral sides in most concentric needle motor unit action potential (MUAP) parameters, such as amplitude (768.7 ± 441.7 vs. 855.0 ± 289.9 μV), duration (8.9 ± 1.8 vs. 8.68 ± 0.9 ms) and size index (1.2 ± 0.5 vs. 1.1 ± 0.3) (*P* > 0.18), nor was there a significant difference in single fiber EMG recorded jitter (37.0 ± 9.6 vs. 39.9 ± 10.6 μs, *P* = 0.45). The increase in FD suggests motor units of the paretic FDI have enlarged due to collateral reinnervation. However, sprouting might be insufficient to result in a statistically significant change in the concentric needle MUAP parameters. Single fiber EMG appears more sensitive than concentric needle EMG to reflect electrophysiological changes in motor units after stroke. Both single fiber and concentric needle EMG recordings may be necessary to better understand muscle changes after stroke, which is important for development of appropriate rehabilitation strategies. The results provide further evidence that motor units are remodeled after stroke, possibly in response to a loss of motoneurons.

## Introduction

Hemiparesis, the reduction in movement capability and strength in the limb opposite the brain lesion (i.e., paretic limb), is a lasting outcome in people who have suffered a cerebrovascular stroke (Colebatch and Gandevia, 1989; Jorgensen et al., 1995a,b; Li, 2017). The loss of hand function is particularly devastating because everyday tasks such as dressing or carrying parcels may be disrupted (Ward, 2011; Kamper et al., 2014; Li et al., 2014a; Lu et al., 2017). The extent of hemiparesis varies from patient to patient, often presumed to reflect the severity and location of the lesion. However, it may also be related to secondary changes in the peripheral motor apparatus, both adaptive and maladaptive (Colebatch and Gandevia, 1989; Klein et al., 2010; Zhou et al., 2013).

Motor units are remodeled following a cerebrovascular lesion. During the initial weeks to months following stroke, there is electrophysiological evidence of motor unit loss in upper and lower muscles of the paretic limb (McComas et al., 1971; Hara et al., 2004). At the same time, there is on-going denervation based on the presence of positive sharp waves and fibrillation potentials recorded by concentric needle EMG (Spaans and Wilts, 1982; Benecke et al., 1983; Brown and Snow, 1990; Dattola et al., 1993; Kouzi et al., 2014). However, some of the denervated fibers are likely reinnervated by collateral sprouting from intact motor units, a demonstration of motor unit remodeling. Hence, muscle fiber density recorded by single fiber EMG is larger in the paretic limb compared to the contralateral limb (Cruz Martinez et al., 1982; Lukács et al., 2009). In some instances, muscle biopsies taken from paretic muscles reveal grouped atrophy and fiber-type grouping, further evidence of on-going denervation and reinnervation processes (Segura and Sahgal, 1981).

The first dorsal interosseous (FDI) is a commonly used model for *in vivo* study of neuromuscular function (Galganski et al., 1993; Kalmar and Cafarelli, 2004; Zhou et al., 2012; Prak et al., 2015; Li et al., 2016). It is the only muscle that abducts the index finger and its nerve supply is easily accessible for clinical EMG testing and assessment of motor unit properties (Young and Mayer, 1982). Because of its importance for hand function, a few have studied FDI properties following stroke (Young and Mayer, 1982; Li et al., 2011, 2013, 2014a,b, 2015, 2016; Zhou et al., 2013; Suresh et al., 2015; Hu et al., 2016). In chronic stroke subjects, there are fewer FDI motor units in the paretic limb compared to the contralateral limb (Li et al., 2011, 2016). In addition, electrical and mechanical properties of remaining intact units are altered. In chronic stroke, the paretic FDI was found to contain more motor units that are both slower contracting and fatigable, possibly indicative of disuse (Young and Mayer, 1982). Others found the motor unit population to contain relatively more very large and small amplitude motor unit action potentials (MUAPs) based on surface EMG recordings (Li et al., 2011, 2016). The explanation for this more heterogeneous mix of different sized MUAP is uncertain, but may reflect varying degrees of denervation, reinnervation, and fiber atrophy.

The single fiber EMG technique identifies the activity of a single muscle fiber and thus the activity of a single motor unit (Ekstedt, 1964; Stalberg, 1966). Two measurements in particular, fiber density (FD) and jitter, are invaluable for enhancing understanding of motor unit (muscle fiber) remodeling. Fiber density reflects the local organization of muscle fibers within the motor unit, and jitter provides an indication of the stability of neuromuscular transmission (Sanders and Stalberg, 1996). Both measures are sensitive indicators of denervation and reinnervation processes associated with disease or injury.

A few investigators have used single fiber EMG to examine FD and jitter post-stroke. In one report, FD of the extensor digitorum communis muscle was determined in 20 persons with hemiplegia lasting from 2 months to 8 years, 16 of whom suffered a stroke (Cruz Martinez et al., 1982). They found that the mean FD was significantly larger in the paretic compared to the contralateral limb (1.97 vs. 1.55), but FD was unrelated to disease duration. Lukács et al. (2009) reported significantly larger FD in the abductor digiti minimi muscle of the paretic limb compared to the contralateral limb in subjects with stroke lasting from 2 weeks to 4 years. They also reported that the paretic to contralateral limb FD ratio increased significantly with disease duration between 2 weeks and 10 months, with no further change beyond 10 months. Chang (1998) reported significantly larger jitter in the extensor digitorum communis and tibialis anterior muscles of 28 stroke subjects compared to 13 healthy control subjects, based on electrical stimulation single fiber EMG. Although motor unit population and control properties of the paretic FDI muscle have been investigated, no one has studied post-stroke FDI motor unit properties using single fiber EMG.

The purpose of this study was to characterize the differences in motor unit and single fiber properties between the paretic and contralateral FDI in a group of chronic stroke subjects. Concentric needle EMG recordings for quantitative MUAP analysis were made together with single fiber EMG measures of FD and jitter to better understand motor unit remodeling following stroke.

## Materials and Methods

### Subjects

Eleven hemiparetic stroke survivors (seven females and four males) aged from 45–79 years (mean: 61.7 ± 10.8 years) volunteered to participate in the study. The inclusion criteria included: (i) an interval of at least 6 months after stroke; (ii) hemiplegia secondary to an ischemic or hemorrhagic stroke; (iii) ability to follow commands and move their index finger; and (iv) ability to sign an informed consent form. All subjects provided written informed consent, which was conducted in accordance with the Declaration of Helsinki and approved by the Committee for the Protection of Human Subjects at the University of Texas Health Science Center at Houston (UTHealth) and TIRR Memorial Hermann Hospital (Houston, United States).

Upper limb motor function of each stroke subject was evaluated using the Fugl-Meyer test and Chedoke-McMaster test (Fugl-Meyer et al., 1975; Gowland et al., 1993). The Fugl-Meyer assessment is a cumulative numerical scoring system encompassing measures of motor function, balance, sensation, and joint range of motion for patients with post-stroke hemiplegia. The Chedoke-McMaster test is an assessment used to determine the severity of physical impairment for clients with stroke and other neurological disorders (only hand function among its 6 dimensions was assessed in this study). Maximal pinch and grip forces were measured bilaterally. A summary of the subjects’ information is presented in Table 1.

**Table 1:** Stroke subjects’ information.

ID	Age (years)	Stroke duration (years)	Paretic side	Fugl-Meyer	Chedoke	Grip force (kg)		Pinch force (kg)	
							
						Paretic	Contra-	Paretic	Contra-
S1	79	14	R	21	3	2.7	26.8	0.3	4
S2	45	6	R	51	3	12.5	33.7	1	7
S3	71	6	R	21	1	8.7	39.0	1.7	10
S4	52	2	L	15	2	3.2	28.1	2	6
S5	64	15	L	24	3	8.3	29.6	3.5	6.2
S6	64	6	R	40	3	7.6	17.3	3.1	5.0
S7	63	1	L	24	2	3.0	33.0	1.7	8.3
S8	51	1	R	27	2	4.5	25.3	1.7	6.1
S9	68	9	R	40	3	5.0	23.6	1.9	4.3
S10	72	10	L	39	3	10.9	42.8	5.5	10.7
S11	50	7	L	24	3	19.3	44.7	4.3	11.2

*Contra-, Contralateral; Stroke duration, the time after stroke onset.*

### Voluntary Muscle Activation Protocol

Subjects were comfortably seated with the forearm of the tested arm pronated and placed on a height-adjustable table. Prior to electrode placement, the skin of the examined regions was cleaned using an alcohol pad. A surface reference electrode was placed over the ulna styloid. Subjects generated voluntary activation of the FDI by abducting their index finger to push a 5-kg weight in a horizontal direction on the table for at least 30 s. Verbal encouragement was provided by the examiner. To avoid muscle fatigue, subjects had sufficient rest after each recording.

### Concentric-Needle EMG

Concentric needle EMG recordings were carried out using a Natus UltraPro S100 electromyographic system (Natus Neurology Inc., Middleton, WI, United States). The recordings were performed using a conventional concentric needle electrode with a diameter of 0.58 mm and a recording area of 0.07 mm^2^ (Alpine bioMed, Denmark). At least 25MUAPs were recorded from each FDI, which required advancing the needle to different depths at each of 3–4 skin incisions. The filter settings ranged from 2–5,000 Hz using a 200 μV/cm gain and a 3 ms/div sweep speed for the multi-MUAP analysis.

Action potentials were automatically classified and analyzed using a built-in multi-MUAP analysis program. The MUAP parameters measured were the following (Stalberg et al., 1996): duration, the time between the starting-point and end-point of the slow component of the MUAP, defined as 20 μV amplitude deviation from baseline; amplitude, the voltage difference between the negative peak and the positive peak within the duration; the number of turns, the number of peaks in the MUAP waveform, based on at least a 100 μV change in amplitude between successive turns; phases, the part of the MUAP that falls between two baseline crossings, excluding baseline crossings due to noise; area, the rectified MUAP within the duration; area/amplitude ratio, expressing the overall “thickness” of an MUAP; and size index, defined as 2 × log10 (amp) + area/amp, which reflects the number of muscle fibers within the needle uptake area (Sonoo and Stalberg, 1993; Kouzi et al., 2014).

### Single Fiber EMG

Single fiber EMG recordings were carried out using a Natus UltraPro S100 electromyographic system. The filter setting for FD and jitter was 500–10,000 Hz.

#### Fiber Density

Recordings were obtained with a single fiber EMG electrode (0.45 mm 26G × 40 mm, Natus). The electrode was inserted into the FDI muscle and slowly advanced while the subject abducted the index finger in an attempt to push the 5 kg weight. The electrode was optimally positioned to maximize the amplitude of a single fiber action potential. After optimization, the number of associated single fiber action potentials time-locked to the triggering potential was determined. The electrode was advanced to a new position in the FDI muscle and the process of counting potentials was repeated. Different depths, at each of 2–3 skin insertions, were studied until at least 20 different recording sites in the mid belly of the FDI muscle had been examined. The fiber density was the mean number of potentials counted in 20 recording sites.

#### Jitter

The single fiber EMG electrode was inserted in the mid belly of the FDI and was slowly advanced so as to record two (or more) time-locked muscle fiber action potentials from the same motor unit during a mild voluntary contraction. The located single fiber action potentials had a brief rise time (< 300 μs) and an amplitude greater than 200 μV, and at least 50 discharges from the motor unit were recorded. The electrode was then advanced to a new position to record 50 discharges from a different motor unit. Different depths, at each of 2–3 skin insertions, were studied until at least 20 different recording sites had been examined. The jitter was calculated as the mean values of the consecutive differences (MCD) of the successive inter-potential intervals, according to the following formula:

$$\text{MCD}=(|{\text{IPI}}_{1}-{\text{IPI}}_{2}|+|{\text{IPI}}_{2}-{\text{IPI}}_{3}|+\ldots +|{\text{IPI}}_{n-1}-{\text{IPI}}_{n}|)/(\text{n}-1)$$

where *IPI*_*i*_ is the inter-potential interval between the two single fiber potentials of the motor unit (Sanders and Stalberg, 1996).

### Statistical Analysis

Statistical analysis was performed using SPSS (SPSS, Inc., 2007, Chicago, IL, United States). The Shapiro-Wilk test was used to test for possible deviations from normality and none was found (*P* > 0.05). The Student’s paired *t*-test was used to investigate side-to-side differences for the examined parameters. Spearman’s correlation was applied to investigate correlations between different EMG parameters and disease chronicity or clinical scores. Differences were considered significant when *P* < 0.05 and data are presented as mean ± standard deviation (SD).

## Results

### Clinical Scores and MVC

Most subjects had significant motor impairment in the upper limb (Table 1). No subject had a Chedoke score above three, which means that any active voluntary movement of the FDI occurred as part of a synergy. Seven of the subjects had a Fugl-Meyer score less than half of the maximum (66) score. The MVC force (kg) of the paretic limb was less than half of the contralateral limb during pinch (2.43 ± 1.53 kg vs. 7.17 ± 2.54 kg, *P* < 0.005) and grip (7.78 ± 5.04 kg vs. 31.27 ± 8.39 kg; *P* < 0.005).

### Concentric Needle EMG

**FIGURE 1:**
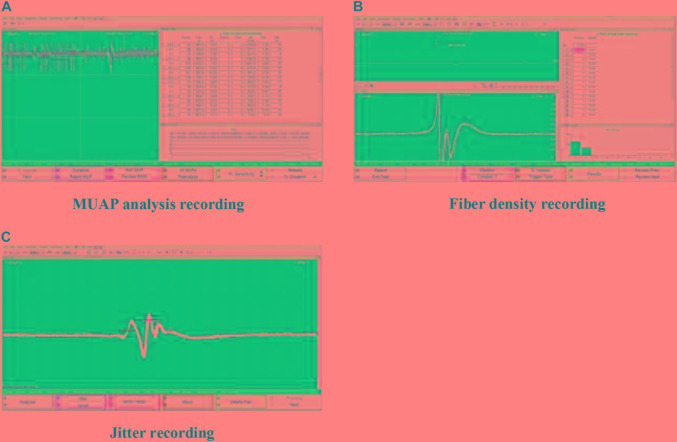
Screenshot during the experiment **(A)** MUAP analysis recording; **(B)** Fiber density recording; and **(C)** Jitter recording.

Bilateral MUAP recordings were performed in all 11 stroke subjects. Figure 1A shows a representative MUAP recorded from the paretic FDI of one subject. The MUAP area/amplitude ratio was significantly larger in the paretic side than the contralateral side (*P* < 0.05, Table 2). Similarly, the MUAP size index was larger in the paretic limb, but the difference was not statistically significant (*P* = 0.18). Unexpectedly, the number of MUAP turns and phases tended to be smaller in the paretic limb, although differences were not significant (*P* = 0.15 and 0.06). The MUAP amplitude and duration did not differ significantly between sides, nor was there a trend for any difference. Disease chronicity (which is expressed as the years post-stroke) was unrelated to the paretic muscle MUAP amplitude (Spearman’s correlation: *r* = 0.49, *P* = 0.12), area (*r* = 0.31, *P* = 0.35), duration (*r* = 0.22, *P* = 0.51), phases (*r* = 0.10, *P* = 0.76), or turns (*r* = 0.12, *P* = 0.71). Similarly, Fugl-Meyer and Chedoke scores were not significantly correlated with MUAP amplitude (Fugl-Meyer: *r* = 0.09, *P* = 0.79; Chedoke: *r* = 0.47, *P* = 0.15), area (Fugl-Meyer: *r* = 0.24, *P* = 0.49; Chedoke: *r* = 0.42, *P* = 0.20), duration (Fugl-Meyer: *r* = 0.16, *P* = 0.65; Chedoke: *r* = 0.19, *P* = 0.57), phases (Fugl-Meyer: *r* = 0.02, *P* = 0.96; Chedoke: *r* = 0.19, *P* = 0.59), or turns (Fugl-Meyer: *r* = 0.10, *P* = 0.78; Chedoke: *r* = 0.24, *P* = 0.47).

**Table 2:** Concentric needle MUAP properties.

Parameter	Paretic side		Contralateral side		*P*-value
			
	Mean ± SD	Min–Max	Mean	Min–Max	
Amplitude (μV)	768.7 ± 441.7	337.5–1936	855.0 ± 289.9	531.3–1462.3	0.49
Duration (ms)	8.9 ± 1.8	7.3–10.9	8.68 ± 0.9	7.7–10.7	0.67
Area (μVms)	1200.2 ± 729.4	307.2–2963.1	1130.3 ± 420.3	591.5–1766.3	0.71
Area/amplitude ratio	1.5 ± 0.3	0.98–1.86	1.4 ± 0.1	1.19–1.41	0.04
Number of turns	2.9 ± 0.3	2.4–3.4	3.0 ± 0.2	2.8–3.3	0.15
Number of phases	2.7 ± 0.3	2.2–3.1	2.9 ± 0.2	2.6–3	0.06
Size index	1.2 ± 0.5	0.6–2.1	1.1 ± 0.3	0.6–1.4	0.18

### Singe Fiber EMG

Bilateral single fiber EMG recordings of FD and jitter were successfully completed in 10 and 9 stroke subjects, respectively. Figures 1B,C show representative recordings of FD and jitter analysis. FD was larger in the paretic than the contralateral limb in 9 of the 10 subjects, and the means were significantly different between sides (1.6 ± 0.2 vs. 1.3 ± 0.1, respectively, *P* = 0.003). However, jitter or MCD was not different between the paretic and contralateral muscles (37.0 ± 9.6 μs vs. 39.9 ± 10.6 μs, respectively, *P* = 0.45). Disease chronicity was unrelated to FD (*r* = 0.31, *P* = 0.39) or MCD (*r* = -0.07, *P* = 0.86), nor was Fugl-Meyer or Chedoke scores correlated to FD (Fugl-Meyer: *r* = 0.51, *P* = 0.13; Chedoke: *r* = -0.14, *P* = 0.71) or MCD (Fugl-Meyer: *r* = 0.33, *P* = 0.43; Chedoke: *r* = -0.10, *P* = 0.83).

## Discussion

### FD and Concentric Needle MUAP

A primary feature of this study was the combination of single fiber EMG and concentric needle EMG recordings to assess the characteristics of voluntarily recruited motor units in chronic stroke subjects. We were able to record FD of the FDI muscle bilaterally in 10 of the 11 stroke subjects, and it was found to be significantly higher in the paretic than the contralateral side, consistent with previous studies of hand (abductor digiti minimi) (Lukács et al., 2009) and forearm (extensor digitorum communis) muscles following stroke (Cruz Martinez et al., 1982). These findings indicate that motor units in muscles of the paretic limb contain more dense groups of muscle fibers than the contralateral limb.

There is evidence of changes in motor unit properties early after stroke. This includes denervation and motor unit loss and subsequent reinnervation of previously denervated fibers (McComas et al., 1971; Hara et al., 2004). The reinnervation likely arises from collateral sprouts from axons of intact motor units, thus resulting in an increase in the number of fibers per motor unit (increased FD). We found that the FD in the paretic FDI was unrelated to stroke duration. This is consistent with a previous stroke study of the abductor digiti minimi, in which FD was not significantly correlated with disease duration in a group of subjects who had stroke for more than 10 months (Lukács et al., 2009). Hence, the FD values in the current study likely reflect a stable condition where motor unit remodeling has been essentially completed.

In contrast to the augmented FD, there was no corresponding increase in the average amplitude, duration, or complexity (number of phases/turns) of concentric needle recorded MUAPs in the paretic FDI. This is consistent with findings in the abductor digiti minimi of acute and chronic stroke subjects, in which there was little to no side-to-side difference in concentric needle MUAP properties (Lukács, 2005; Kouzi et al., 2014). However, the paretic abductor digiti minimi did contain a larger number of outliers for MUAP amplitude and MUAP duration, both of which were significantly related to stroke duration and stroke severity (Lukács, 2005). It is noteworthy that the average time after stroke onset was 5.3 months in the study by Lukács (2005) and 6.3 years in the present study. Because the concentric needle electrode records from a larger motor unit territory compared to single fiber EMG, the recorded action potentials may be impacted more by changes in the various motor unit components (i.e., motoneuron, neuromuscular junction, muscle fiber) that develop chronically after stroke. Hence, the lack of MUAP changes recorded by concentric needle EMG and other methods (i.e., *F*-wave) (Hara et al., 2004), could reflect greater muscle fiber atrophy in paretic muscles of chronic patients (Scelsi et al., 1984).

Motor unit remodeling is a normal part of the aging process (Hepple and Rice, 2016). There is evidence of denervation and motor unit loss and increased size of the surviving motor units through collateral sprouting. Most of these changes become pronounced beyond the eighth decade of age. It is uncertain whether the larger mean FD in the paretic FDI reported here was exacerbated by aging. Only three of the subjects were in their eight decade of age (71, 72, and 79 years), having suffered a stroke 6, 10, and 14 years prior to the study, respectively (Table 1). In a previous cross-sectional study of 59 stroke subjects (37–78 years, mean age 61 years), FD of the abductor digiti minimi increased progressively with increased time since stroke during the first 10 months (*n* = 13), after which it was stable. It is uncertain whether the age of the 13 subjects differed compared to the other 46 subjects. The impact of aging on post-stroke motor unit properties remains an important area for further study.

### Jitter

Jitter, which assesses the integrity of neuromuscular transmission (Sanders and Stalberg, 1996) has received little study in stroke (Cruz Martinez et al., 1982; Chang, 1998). We recorded jitter in 9 subjects, the first such demonstration in the FDI muscle after stroke. The MCD was found to be not significantly different between the paretic and contralateral FDI muscles, consistent with findings in the paretic extensor digitorum communis recorded in a few patients (Cruz Martinez et al., 1982). In that study, none of the potentials showed increased jitter in patients with chronic stroke (>10 months), whereas some potentials showed increased jitter and occasional intermittent impulse blocking in patients with disease duration less than 9 months (Cruz Martinez et al., 1982). However, these results conflict with a study of stimulated single fiber EMG jitter in paretic muscles of 28 stroke subjects, who had stroke between 2 months and 8 years, and muscles of 13 healthy controls (Chang, 1998). In both the extensor digitorum communis and tibialis anterior, the MCD was significantly larger in the paretic muscles, progressively increased with stroke duration, and was larger in severely weak compared to moderately weak muscles (Chang, 1998). This result suggests that neuromuscular transmission in paretic muscles is unstable, whereas the present result indicates no such change. The explanation for this discrepancy is uncertain, but could be related to differences in the method (stimulated vs. voluntary induced singe fiber EMG) and/or the muscle studied.

### Weakness and Motor Unit Remodeling

Maximal pinch and grip strength of the paretic limb was reduced by more than one-half, and the FDI muscle was likely similarly weak (Zhou et al., 2013). Muscle atrophy is unlikely to be a major contributor to weakness given that the average atrophy in the FDI in chronic stroke is modest (∼13%, Triandafilou and Kamper, 2012). Conceivably, the reinnervation demonstrated here prevented more severe muscle atrophy that would otherwise occur from motor unit loss (Li et al., 2016). Still, much of the weakness is likely due to reduced central nervous system activation of muscle. Hence, some motor units may not have been recruited, whereas other units likely fired at a lower rate, despite maximum effort (Chou et al., 2013; McNulty et al., 2014; Li et al., 2015; Hu et al., 2016). Further work is necessary to better understand the relative contributions of impaired motor unit activation and motor unit remodeling on hand weakness after stroke.

We did not record motor unit properties from a group of healthy controls, a limitation of the current study. Our reported limb difference in FD could in theory be explained by a reduction in the contralateral limb FD below normal rather than an actual increase in FD of the paretic limb. However, this is unlikely the case, as there is no obvious explanation as to how this would come about. Furthermore, previous studies found that FD of the paretic limb was significantly larger than the limb of healthy controls (Lukács et al., 2009). Another limitation is the relatively small sample size. It is possible that the limb differences in FD would have been related to disease duration or clinical motor function in a larger sample of stroke subjects (Lukács et al., 2009).

### Conclusion

Single fiber EMG and concentric needle EMG were applied to examine the FDI muscle bilaterally in eleven hemiparetic stroke subjects. A significant increase in FD was found in the paretic muscle compared with the contralateral side. However, no statistically significant difference was found between the paretic and unaffected sides in MUAP amplitude, duration, area, size index, the number of MUAP phases and turns, and jitter. Some of the present results are consistent with earlier findings (Cruz Martinez et al., 1982; Lukács et al., 2009) and provide new evidence of motor unit remodeling in the FDI in chronic stroke subjects. Our findings suggest that single fiber EMG is more sensitive than concentric needle EMG in reflecting changes in motor unit size or FD in people with chronic stroke.

## Author Contributions

BY performed data collection, analysis and interpretation, and wrote the first draft of the manuscript. CK participated in study design, data analysis, interpretation, and manuscript writing. HH participated in data collection and analysis. SL participated in study design, data analysis, interpretation, and revised the manuscript. PZ performed study design and oversaw data collection, analysis and interpretation of the study, and revised the manuscript. All the authors read and approved the final manuscript.

## Conflict of Interest Statement

The authors declare that the research was conducted in the absence of any commercial or financial relationships that could be construed as a potential conflict of interest.

## References

[B1] Benecke R. Berthold A. Conrad B. (1983). Denervation activity in the EMG of patients with upper motor neuron lesions: time course, local distribution and pathogenetic aspects. *J. Neurol.* 230 143–151. 10.1007/BF00313625 6197509

[B2] Brown W. F. Snow R. (1990). Denervation in hemiplegic muscles. *Stroke* 21 1700–1704. 10.1161/01.STR.21.12.1700 2264077

[B3] Chang C. W. (1998). Evident trans-synaptic degeneration of motor neurons after stroke: a study of neuromuscular jitter by axonal microstimulation. *Electroencephalogr. Clin. Neurophysiol.* 109 199–202. 10.1016/S0924-980X(98)00011-3 9741785

[B4] Chou L. W. Palmer J. A. Binder-Macleod S. Knight C. A. (2013). Motor unit rate coding is severely impaired during forceful and fast muscular contractions in individuals post stroke. *J. Neurophysiol.* 109 2947–2954. 10.1152/jn.00615.2012 23554434 PMC3680820

[B5] Colebatch J. G. Gandevia S. C. (1989). The distribution of muscular weakness in upper motor neuron lesions affecting the arm. *Brain* 112(Pt 3), 749–763. 10.1093/brain/112.3.749 2731028

[B6] Cruz Martinez A. del Campo F. Mingo M. R. Perez Conde M. C. (1982). Altered motor unit architecture in hemiparetic patients. A single fibre EMG study. *J. Neurol. Neurosurg. Psychiatry.* 45 756–757. 10.1136/jnnp.45.8.756 7131005 PMC1083175

[B7] Dattola R. Girlanda P. Vita G. Santoro M. Roberto M. L. Toscano A. (1993). Muscle rearrangement in patients with hemiparesis after stroke: an electrophysiological and morphological study. *Eur. Neurol.* 33 109–114. 10.1159/000116915 8467816

[B8] Ekstedt J. (1964). Human single muscle fiber action potentials. Extracellular recording during voluntary and chemical activation with some comments on end-plate physiology and on the fiber arrangement of the motor unit. *Acta. Physiol. Scand. Suppl.* 61(Suppl. 226), 1–96. 14150641

[B9] Fugl-Meyer A. R. Jaasko L. Leyman I. Olsson S. Steglind S. (1975). The post-stroke hemiplegic patient. I: a method for evaluation of physical performance. *Scand. J. Rehabil. Med.* 7 13–31. 1135616

[B10] Galganski M. E. Fuglevand A. J. Enoka R. M. (1993). Reduced control of motor output in a human hand muscle of elderly subjects during submaximal contractions. *J. Neurophysiol.* 69 2108–2115. 10.1152/jn.1993.69.6.2108 8350134

[B11] Gowland C. Stratford P. Ward M. Moreland J. Torresin W. Van Hullenaar S. (1993). Measuring physical impairment and disability with the Chedoke-McMaster Stroke Assessment. *Stroke* 24 58–63. 10.1161/01.STR.24.1.58 8418551

[B12] Hara Y. Masakado Y. Chino N. (2004). The physiological functional loss of single thenar motor units in the stroke patients: when does it occur? Does it progress? *Clin. Neurophysiol.* 115 97–103. 10.1016/j.clinph.2003.08.002 14706475

[B13] Hepple R. T. Rice C. L. (2016). Innervation and neuromuscular control in ageing skeletal muscle. *J. Physiol.* 594 1965–1978. 10.1113/JP270561 26437581 PMC4933121

[B14] Hu X. Suresh A. K. Rymer W. Z. Suresh N. L. (2016). Altered motor unit discharge patterns in paretic muscles of stroke survivors assessed using surface electromyography. *J. Neural. Eng.* 13 046025. 10.1088/1741-2560/13/4/046025 27432656 PMC5439147

[B15] Jorgensen H. S. Nakayama H. Raaschou H. O. Vive-Larsen J. Stoier M. Olsen T. S. (1995a). Outcome and time course of recovery in stroke. part I: outcome. the copenhagen stroke study. *Arch. Phys. Med. Rehabil.* 76 399–405. 7741608 10.1016/s0003-9993(95)80567-2

[B16] Jorgensen H. S. Nakayama H. Raaschou H. O. Vive-Larsen J. Stoier M. Olsen T. S. (1995b). Outcome and time course of recovery in stroke. part II: time course of recovery. the copenhagen stroke study. *Arch. Phys. Med. Rehabil.* 76 406–412. 7741609 10.1016/s0003-9993(95)80568-0

[B17] Kalmar J. M. Cafarelli E. (2004). Central fatigue and transcranial magnetic stimulation: effect of caffeine and the confound of peripheral transmission failure. *J. Neurosci. Methods.* 138 15–26. 10.1016/j.jneumeth.2004.03.006 15325107

[B18] Kamper D. G. Fischer H. C. Conrad M. O. Towles J. D. Rymer W. Z. Triandafilou K. M. (2014). Finger-thumb coupling contributes to exaggerated thumb flexion in stroke survivors. *J. Neurophysiol.* 111 2665–2674. 10.1152/jn.00413.2013 24671534 PMC6442660

[B19] Klein C. S. Brooks D. Richardson D. McIlroy W. E. Bayley M. T. (2010). Voluntary activation failure contributes more to plantar flexor weakness than antagonist coactivation and muscle atrophy in chronic stroke survivors. *J. Appl. Physiol.* 109 1337–1346. 10.1152/japplphysiol.00804.2009 20724561

[B20] Kouzi I. Trachani E. Anagnostou E. Rapidi C. A. Ellul J. Sakellaropoulos G. C. (2014). Motor unit number estimation and quantitative needle electromyography in stroke patients. *J. Electromyogr. Kinesiol.* 24 910–916. 10.1016/j.jelekin.2014.09.006 25304197

[B21] Li S. (2017). Spasticity, motor recovery, and neural plasticity after stroke. *Front. Neurol.* 8:120 10.3389/fneur.2017.00120 PMC5377239 28421032

[B22] Li X. Fisher M. Rymer W. Z. Zhou P. (2016). Application of the f-response for estimating motor unit number and amplitude distribution in hand muscles of stroke survivors. *IEEE Trans. Neural. Syst. Rehabil. Eng.* 24 674–681. 10.1109/TNSRE.2015.2453274 26168437 PMC4902775

[B23] Li X. Holobar A. Gazzoni M. Merletti R. Rymer W. Z. Zhou P. (2015). Examination of poststroke alteration in motor unit firing behavior using high-density surface emg decomposition. *IEEE Trans. Biomed. Eng.* 62 1242–1252. 10.1109/TBME.2014.2368514 25389239 PMC4406795

[B24] Li X. Liu J. Li S. Wang Y. C. Zhou P. (2014a). Examination of hand muscle activation and motor unit indices derived from surface EMG in chronic stroke. *IEEE Trans. Biomed. Eng.* 61 2891–2898. 10.1109/TBME.2014.2333034 24967982 PMC4251955

[B25] Li X. Shin H. Zhou P. Niu X. Liu J. Rymer W. Z. (2014b). Power spectral analysis of surface electromyography (EMG) at matched contraction levels of the first dorsal interosseous muscle in stroke survivors. *Clin. Neurophysiol.* 125 988–994. 10.1016/j.clinph.2013.09.044 24268816

[B26] Li X. Suresh A. Zhou P. Rymer W. Z. (2013). Alterations in the peak amplitude distribution of the surface electromyogram poststroke. *IEEE Trans. Biomed. Eng.* 60 845–852. 10.1109/TBME.2012.2205249 22736632

[B27] Li X. Wang Y. C. Suresh N. L. Rymer W. Z. Zhou P. (2011). Motor unit number reductions in paretic muscles of stroke survivors. *IEEE Trans. Inf. Technol. Biomed.* 15 505–512. 10.1109/TITB.2011.2140379 21478079

[B28] Lu Z. Tong K. Y. Shin H. Li S. Zhou P. (2017). Advanced Myoelectric Control For Robotic Hand-Assisted Training: Outcome From A Stroke Patient. *Front. Neurol.* 8:107. 10.3389/fneur.2017.00107 28373860 PMC5357829

[B29] Lukács M. (2005). Electrophysiological signs of changes in motor units after ischaemic stroke. *Clin. Neurophysiol.* 116 1566–1570. 10.1016/j.clinph.2005.04.005 15905127

[B30] Lukács M. Vecsei L. Beniczky S. (2009). Changes in muscle fiber density following a stroke. *Clin. Neurophysiol.* 120 1539–1542. 10.1016/j.clinph.2009.06.001 19564129

[B31] McComas A. J. Sica R. E. Upton A. R. Aguilera N. Currie S. (1971). Motoneurone dysfunction in patients with hemiplegic atrophy. *Nat. New Biol.* 233 21–23. 10.1038/newbio233021a0 5286222

[B32] McNulty P. A. Lin G. Doust C. G. (2014). Single motor unit firing rate after stroke is higher on the less-affected side during stable low-level voluntary contractions. *Front. Hum. Neurosci.* 17:518. 10.3389/fnhum.2014.00518 25100969 PMC4102083

[B33] Prak R. F. Doestzada M. Thomas C. K. Tepper M. Zijdewind I. (2015). Reduced voluntary drive during sustained but not during brief maximal voluntary contractions in the first dorsal interosseous weakened by spinal cord injury. *J. Appl. Physiol.* 119 1320–1329. 10.1152/japplphysiol.00399.2015 26404618 PMC4669347

[B34] Sanders D. B. Stalberg E. V. (1996). AAEM minimonograph #25: single-fiber electromyography. *Muscle Nerve* 19 1069–1083. 10.1002/(SICI)1097-4598(199609)19:9<1069::AID-MUS1>3.0.CO;2-Y 8761262

[B35] Scelsi R. Lotta S. Lommi G. Poggi P. Marchetti C. (1984). Hemiplegic atrophy. Morphological findings in the anterior tibial muscle of patients with cerebral vascular accidents. *Acta. Neuropathol.* 62 324–331. 10.1007/BF00687615 6730908

[B36] Segura R. P. Sahgal V. (1981). Hemiplegic atrophy: electrophysiological and morphological studies. *Muscle Nerve* 4 246–248. 10.1002/mus.880040312 7242561

[B37] Sonoo M. Stalberg E. (1993). The ability of MUP parameters to discriminate between normal and neurogenic MUPs in concentric EMG: analysis of the MUP “thickness” and the proposal of “size index”. *Electroencephalogr. Clin. Neurophysiol.* 89 291–303. 10.1016/0168-5597(93)90068-Z 7691568

[B38] Spaans F. Wilts G. (1982). Denervation due to lesions of the central nervous system. *An EMG study in cases of cerebral contusion and cerebrovascular accidents*. *J. Neurol. Sci.* 57 291–305. 10.1016/0022-510X(82)90036-3 7161623

[B39] Stalberg E. (1966). Propagation velocity in human muscle fibers in situ. *Acta. Physiol. Scand. Suppl.* 287 1–112. 5958263

[B40] Stalberg E. Nandedkar S. D. Sanders D. B. Falck B. (1996). Quantitative motor unit potential analysis. *J. Clin. Neurophysiol.* 13 401–422. 10.1097/00004691-199609000-00004 8897206

[B41] Suresh N. L. Concepcion N. S. Madoff J. Rymer W. Z. (2015). Anomalous EMG-force relations during low-force isometric tasks in hemiparetic stroke survivors. *Exp. Brain Res.* 233 15–25. 10.1007/s00221-014-4061-3 25224701

[B42] Triandafilou K. M. Kamper D. G. (2012). Investigation of hand muscle atrophy in stroke survivors. *Clin. Biomech.* 27 268–272. 10.1016/j.clinbiomech.2011.10.002 22033224 PMC3299934

[B43] Ward N. (2011). Assessment of cortical reorganisation for hand function after stroke. *J. Physiol.* 589 5625–5632. 10.1113/jphysiol.2011.220939 22063630 PMC3249038

[B44] Young J. L. Mayer R. F. (1982). Physiological alterations of motor units in hemiplegia. *J. Neurol. Sci.* 54 401–412. 10.1016/0022-510X(82)90203-9 7097310

[B45] Zhou P. Li X. Rymer W. Z. (2012). Computing motor unit number index of the first dorsal interosseous muscle with two different contraction tasks. *Med. Eng. Phys.* 34 1209–1212. 10.1016/j.medengphy.2012.06.011 22818404 PMC3514832

[B46] Zhou P. Li X. Rymer W. Z. (2013). EMG-force relations during isometric contractions of the first dorsal interosseous muscle after stroke. *Top. Stroke Rehabil.* 20 537–544. 10.1310/tsr2006-537 24273301

